# Neurobehavioral Correlates of Surprisal in Language Comprehension: A Neurocomputational Model

**DOI:** 10.3389/fpsyg.2021.615538

**Published:** 2021-02-11

**Authors:** Harm Brouwer, Francesca Delogu, Noortje J. Venhuizen, Matthew W. Crocker

**Affiliations:** Department of Language Science and Technology, Saarland University, Saarbrücken, Germany

**Keywords:** event-related potentials (ERPs), N400, P600, language comprehension, surprisal theory

## Abstract

Expectation-based theories of language comprehension, in particular Surprisal Theory, go a long way in accounting for the behavioral correlates of word-by-word processing difficulty, such as reading times. An open question, however, is in which component(s) of the Event-Related brain Potential (ERP) signal Surprisal is reflected, and how these electrophysiological correlates relate to behavioral processing indices. Here, we address this question by instantiating an explicit neurocomputational model of incremental, word-by-word language comprehension that produces estimates of the N400 and the P600—the two most salient ERP components for language processing—as well as estimates of “comprehension-centric” Surprisal for each word in a sentence. We derive model predictions for a recent experimental design that directly investigates “world-knowledge”-induced Surprisal. By relating these predictions to both empirical electrophysiological and behavioral results, we establish a close link between Surprisal, as indexed by reading times, and the P600 component of the ERP signal. The resultant model thus offers an integrated neurobehavioral account of processing difficulty in language comprehension.

## 1. Introduction

In language comprehension, an interpretation is incrementally constructed on a more or less word-by-word basis, where some words incur more processing difficulty than others. Expectation-based theories of comprehension, in particular Surprisal Theory (Hale, 2001, 2003; Levy, 2008), have become influential in explaining word-by-word processing difficulty. Surprisal Theory asserts that the effort incurred by a word is proportional to its expectancy in context: difficulty(*w*_*t*_) ≈ −log *P*(*w*_*t*_|*w*_1_ … *w*_*t*−1_, context), where context denotes the extra-sentential context. Indeed, Surprisal estimates derived from language models go a long way in accounting for behavioral correlates of processing difficulty, in particular reading times (e.g., Boston et al., 2008; Demberg and Keller, 2008; Smith and Levy, 2008, 2013; Frank, 2009; Roark et al., 2009; Brouwer et al., 2010). As such, a natural, yet thus far unanswered question is: What are the electrophysiological indices of Surprisal? More specifically, what component(s) of the Event-Related brain Potential (ERP) signal index(es) Surprisal, and what is their relationship to behavioral indices of processing difficulty?

While previous work has sought to answer this question by correlating Surprisal estimates derived from language models with the amplitude of relevant ERP components on a word-by-word basis (Frank et al., 2015), we here take a different approach. Specifically, we build upon two recent computational models of incremental, word-by-word language comprehension. The first is the model of “comprehension-centric” Surprisal by Venhuizen et al. (2019a) that goes beyond typical language models in that Surprisal is derived directly from the interpretations that are constructed during comprehension—rich, probabilistic representations instantiating situation models—thereby rendering it sensitive both to linguistic experience (like language models), but crucially, also to knowledge about the world, which enables the model to also account for “world knowledge”-driven effects on processing (e.g., Albrecht and O'Brien, 1993; Morris, 1994; Myers and O'Brien, 1998; Cook and Myers, 2004; Knoeferle et al., 2005; van Berkum et al., 2005, among others). We here employ these meaning representations in a neurocomputational model by Brouwer et al. (2017) that instantiates the Retrieval-Integration account of the electrophysiology of language comprehension (Brouwer et al., 2012; Brouwer and Hoeks, 2013), thereby offering a mechanistic account of the modulation pattern of the N400 and the P600—the two most salient ERP components for language processing—that explains key data on semantic processing (as reviewed in Kuperberg, 2007; Bornkessel-Schlesewsky and Schlesewsky, 2008; Brouwer et al., 2012).

The resultant model produces, on a word-by-word basis, estimates of the N400, reflecting the contextualized retrieval of word meaning, estimates of the P600, reflecting the integration of retrieved word meaning into the unfolding utterance interpretation, as well as estimates of “comprehension-centric” Surprisal, reflecting the likelihood of a change in interpretation. Critically, while both retrieval and integration are predicted to be sensitive to a notion of expectation, retrieval processes are modulated by the expectancy of *word meaning*, while integration processes are modulated by the expectancy of *utterance meaning*. In order to identify how “comprehension-centric” Surprisal, taken to be indexed by reading times, relates to electrophysiological indices, we require empirical evidence that bears upon these different types of expectancy.

A recent study by Delogu et al. (2019), henceforth DBC, employs a context manipulation design in which they manipulated word meaning expectancy (retrieval/N400) through semantic association (henceforth *association*), and utterance meaning expectancy (integration/P600) through *plausibility*. More specifically, they manipulated the association and plausibility of a target word in German mini-discourses, across three conditions:

**Baseline**                        [+plausible, +associated]
Johann betrat das Restaurant. Wenig später öffnete er die Speisekarte und […]
“*John entered the restaurant. Before long, he opened the menu and […]”*
**Event-related**               [−plausible, +associated]
Johann verließ das Restaurant. Wenig später öffnete er die Speisekarte und […]
“*John left the* restaurant*. Before long, he opened the menu and […]*”
**Event-unrelated**          [−plausible, −associated]
Johann betrat die Wohnung. Wenig später öffnete er die Speisekarte und […]
“*John entered the apartment. Before long, he opened the menu and […]*”

**Figure 1:**
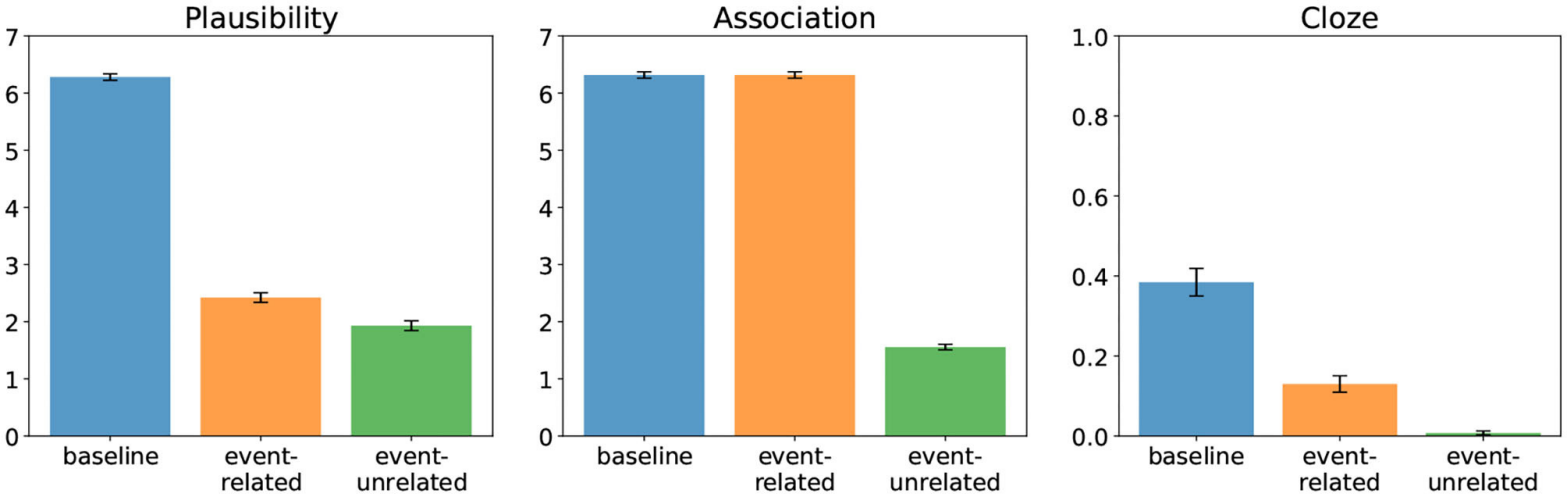
Offline ratings from Delogu et al. (2019) for plausibility **(left)** and association **(middle)**, and estimated Cloze probability of the target **(right)** in all three conditions.

Figure 1 shows the plausibility judgments (left) and association ratings (middle) found by DBC. In both the event-related and the event-unrelated condition, the target word (e.g., “Speisekarte”/“menu”) rendered the entire mini-discourse implausible relative to baseline. In addition, there was also a difference in plausibility between the event-related and event-unrelated condition. Further, the event-related and the event-unrelated conditions differed in the degree of association between the target word and its prior context; that is, in the event-unrelated condition the target word is unassociated with the context, while in the event-related (and baseline) condition it is associated with the context. Figure 1 (right) shows the Cloze probabilities of the target words in all three conditions, as determined based on completions of two-sentence discourses up to and including the determiner preceding the target word. Crucially, the Cloze probabilities—which quantify the expectancy of the critical words in context, and the negative logarithm of which determines their Surprisal—show a qualitatively similar pattern to the plausibility ratings with all conditions differing from each other.

In what follows, we will first derive an explicit neurocomputational model of comprehension that produces explicit N400, P600, and Surprisal estimates for these conditions. Subsequently, we will outline the predictions of the model, the ERP results obtained by DBC, as well as the reading time results from replication of this study using a self-paced reading (SPR) paradigm. Our results suggest a strong qualitative link between “comprehension-centric” Surprisal, as indexed by reading times, and the integration processes underlying the P600 component of the ERP signal. While this conclusion differs from previous findings linking Surprisal to the N400 component, we discuss how these results can be reconciled within the Retrieval-Integration framework, thereby offering a more integrated neurobehavioral account of processing difficulty in language comprehension.

## 2. A Neurocomputational Model

To model both estimates of ERP components (N400 and P600), as well as estimates of Surprisal (reading times), we start from the neurocomputational model of the N400 and P600 by Brouwer et al. (2017), and augment it with the rich, probabilistic situation model representations used by Venhuizen et al. (2019a). Critically, by replacing the thematic role assignment representations used in Brouwer et al. (2017) with these richer meaning representations—which naturally capture probabilistic knowledge about the world—the resultant model produces N400, P600, and “comprehension-centric” Surprisal estimates on a word-by-word basis.

### 2.1. Architecture

The neurocomputational model of language electrophysiology by Brouwer et al. (2017) instantiates the Retrieval-Integration (RI) account of the N400 and the P600 (Brouwer et al., 2012; Brouwer and Hoeks, 2013; Delogu et al., 2019). The RI account postulates that incremental, word-by-word comprehension proceeds in cycles consisting of the Retrieval of word meaning, the ease of which is reflected in N400 amplitude (retrieval of word meaning is facilitated if it is expected given the preceding context), and the subsequent Integration of this word meaning into the unfolding utterance representation, the effort incurred by which is indexed by P600 amplitude (integration difficulty increases as a function of the degree to which integrating retrieved word meaning renders the interpretation unexpected, unclear, or implausible).

Mechanistically, the processing of a word can be conceptualized as a function *process*, which maps an acoustically or orthographically perceived word *w*_*t*_ (word form), and the context as established after processing words *w*_1_ … *w*_*t*−1_ (utterance context), onto an utterance interpretation spanning words *w*_1_ … *w*_*t*_ (utterance representation):

$$\begin{array}{l} process:\;(word\;form,\;utterance\;context)\to utterance\\ \;representation \end{array}$$

This mapping is, however, indirect in that the *process* function is itself composed of a *retrieve* and an *integrate* function, which are hypothesized to underlie the N400 and the P600 components, respectively. The *retrieve* function maps the incoming word form *w*_*t*_ onto a representation of its meaning (word meaning), while taking into account the context in which it occurs (utterance context):

$$\begin{array}{l} retrieve:\;(word\;form,\;utterance\;context)\to word\;meaning\;\\ \;[\sim N400] \end{array}$$

The result of this *retrieve* function (word meaning) serves as input for the *integrate* function, which maps the meaning of *w*_*t*_ (word meaning) and its prior context (utterance context) onto an updated utterance interpretation (utterance representation):

$$\begin{array}{l} integrate:\;(word\;meaning,\;utterance\;context)\to utterance\\ \;representation\;[\sim P600] \end{array}$$

The resultant, updated interpretation determines the context for the retrieval and integration of a next word.

**Figure 2:**
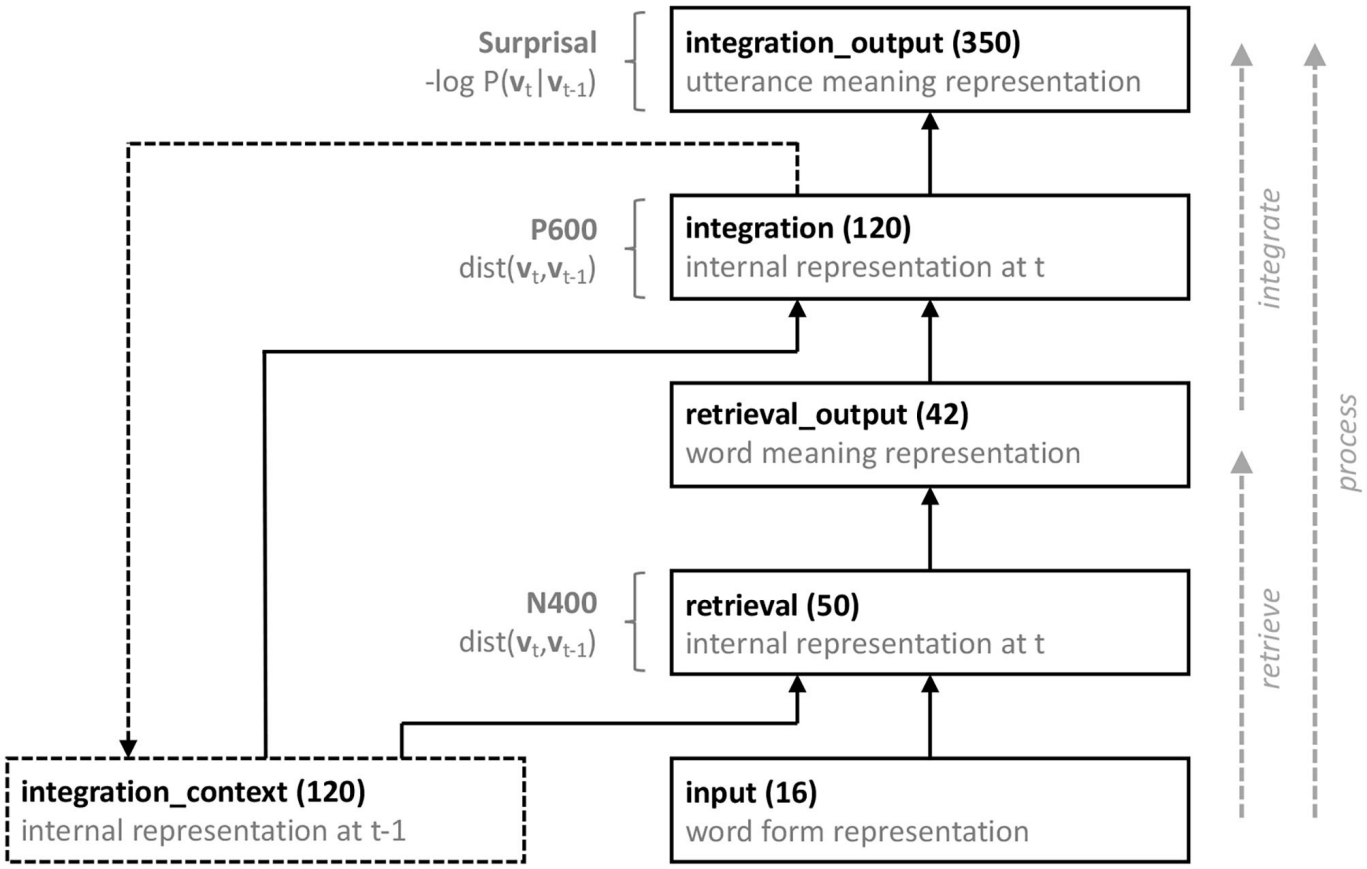
Schematic illustration of the neurocomputational model. Each rectangle represents a layer of artificial (leaky rectified linear) neurons, and each solid arrow represents full connectivity between each neuron in a projecting layer and each neuron in a receiving layer. The dashed rectangle is a context layer, and the dashed arrow represents a copy projection, such that prior to feed-forward propagation the **integration_output** layer receives a copy of the **integration** layer. All groups except the **input** and **integration_context** layer also receive input from a bias unit (not shown). See text for details.

Formally, the neurocomputational model is a recurrent, artificial neural network model that instantiates the *process* function, broken down into its *retrieve* and *integrate* sub-processes. Figure 2 provides a schematic overview of the model architecture. The model consists of five layers of artificial neurons, implementing the input to the model (**input**), a Retrieval module (**retrieval** and **retrieval_output**), and an Integration module (**integration** and **integration_output**). As artificial neurons, we used leaky rectified linear units, the activation function of which is defined as follows (for the leak parameter we used α = 0.3):

(1) $$f(x)=\{\begin{array}{ll} x & \text{if }x>0\\ \alpha x & \text{otherwise} \end{array}$$

Units in the **retrieval_output** and **integration_output** are capped at 1.0—i.e., *f*′(*x*) = *min*(*f*(*x*), 1.0)—as the representations that the model is trained to recover at these layers are binary representations (see below). To facilitate learning, however, units are not capped at zero, allowing a small positive gradient for inactive units.

Time in the model is discrete, and at each processing timestep *t*, activation flows from the **input** layer, through the **retrieval** layer to the **retrieval_output** layer, and from **retrieval_output** layer through the **integration** layer to the **integration_output** layer. To allow for context-sensitive retrieval and integration, the **retrieval** and the **integration** layer both also receive input from the activation pattern in the **integration** layer as established at the previous timestep *t* − 1, effectuated through an additional context layer (**integration_context**; see Elman, 1990). Prior to feed-forward propagation of activation from the **input** to the **integration_output** layer, this **integration_context** layer receives a copy of the **integration** layer (at timestep *t* = 0, the activation value of each unit in the **integration_context** layer is set to 0.5). Finally, all layers except the **input** and **integration_context** layer also receive input from a bias unit, the activation value of which is always 1.

As will be detailed below, the model is trained to incrementally, on a word-by-word basis, map sequences of (orthographic or acoustic) word forms, presented at the **input** layer, onto an utterance meaning representation at the **integration_output** layer, thus instantiating the *process* function at each time tick. Crucially, the mapping from word forms onto an utterance representation is not direct; it is broken down into the *retrieve* and *integrate* sub-processes. Provided a localist representation of an incoming word *w*_*t*_ (**input**), encoding its perceived orthographic/acoustic form, and the unfolding context (**integration_context**), the **retrieval** layer serves to activate a word meaning representation of *w*_*t*_ in the **retrieval_output** layer. Hence, the function of the **retrieval** layer is to retrieve word meaning representations, which take the form of distributed, binary semantic feature vectors (derived from the training sentences using the Correlated Occurrence Analogue to Lexical Semantics, COALS, Rohde et al., 2005; see below). The effort involved in retrieval is taken to be reflected in the N400 amplitude, which is estimated as the degree to which the activation pattern of the **retrieval** layer changes as a result of processing the incoming word:

(2) $$N400(w_{t})=dist(retrieval_{t},retrieval_{t-1})$$

where dist(*x, y*) = 1.0 − cos(*x, y*)^1^. The logic is that if the model finds itself in a state in which the meaning of an incoming word is expected, there will be little change in **retrieval** layer from *t* − 1 to *t*, and the estimated N400 amplitude will be small. If, on the other hand, the meaning of an incoming word is unexpected, this will induce a larger change, and a larger estimated N400 amplitude.

The **integration** layer, in turn, combines the retrieved word meaning representation (**retrieval_output**) with the unfolding utterance context (**integration_context**), into an updated utterance representation (**integration_output**). The **integration** layer thus serves to integrate word meaning into the unfolding interpretation. The effort involved in updating the interpretation with the meaning contributed by the incoming word is taken to be reflected in the P600 amplitude, which is estimated as the degree to which the activation pattern of the **integration** layer changes from *t* − 1 to *t*:

(3) $$\begin{array}{l} \text{P600(}w_{t}\text{)}=\text{dist}\left({\text{integration}}_{t},{\text{integration}}_{t-1}\right) \end{array}$$

where again dist(*x, y*) = 1.0 − cos(*x, y*). If the interpretation is expected, given the linguistic experience of the model and/or its knowledge about the world, integration of the meaning contributed by the incoming word should be relatively effortless, and hence induce a relatively small change in the **integration** layer, thus producing a small estimated P600 amplitude. Conversely, if the interpretation is unexpected, the change in the **integration** layer will be larger, and so will the estimated P600 amplitude.

The utterance meaning representations that the model produces—at its **integration_output** layer—are rich “situation model”-like meaning representations that encode meaning as points in a Distributed Situation-state Space (DSS; Frank et al., 2003, 2009; for a recent reconceptualization of these representations grounded in formal semantics, see Venhuizen et al., 2019c). DSS offers distributed representations that allow for encoding world knowledge, and that are both compositional and probabilistic (see section 2.2.3 below for more detail). Crucially, the probabilistic nature of the DSS representations allows for deriving Surprisal estimates directly from the meaning vectors (Frank and Vigliocco, 2011). In particular, Venhuizen et al. (2019a) define an online, comprehension-centric notion of Surprisal that is sensitive to both linguistic experience and world knowledge, and that derives directly from a change in interpretation from time-step *t* − 1 to *t*:

(4) $$\begin{array}{l} \text{Surprisal(}w_{t}\text{)}=\\ -\log\text{P}\left({\text{integration\_output}}_{t}|{\text{integration\_output}}_{t-1}\right) \end{array}$$

That is, the more likely the interpretation at *t* given the interpretation at *t* − 1, the lower the Surprisal induced by word *w*_*t*_ (see Venhuizen et al., 2019b, for a similar DSS-derived conceptualization of Entropy).

To summarize, the model processes utterances on an incremental word-by-word basis, and produces N400, P600, and Surprisal estimates for every word. More specifically, for a given incoming word form (**input**), and a given context (**integration_context**), the **retrieval** layer retrieves a word meaning representation (**retrieval_output**). Ease of retrieval is reflected in the estimated N400 amplitude. Subsequently, the **integration** layer serves to integrate this retrieved word meaning representation into the unfolding utterance meaning representation (**integration_context**), to produce an updated utterance interpretation (**integration_output**). Ease of integration is reflected in the estimated P600 amplitude, and Surprisal estimates reflect the likelihood of the updated interpretation given the previous interpretation. The model thus predicts a strong correlation between the P600 and Surprisal.

### 2.2. Representations

#### 2.2.1. Word Form Representations

The acoustic/orthographic word form for each of the unique words in the training set is represented as a 16-dimensional localist representation, such that each unit uniquely identifies a single word.

#### 2.2.2. Word Meaning Representations

In line with influential theories of word meaning (see McRae et al., 2005, for a review), our model employs feature-based semantic representations as word meaning representations, in which related concepts may share semantic features. Specifically, like in the Brouwer et al. (2017) model, the semantics associated with individual words are distributed, binary feature-vectors derived using the Correlated Occurrence Analogue to Lexical Semantics (COALS; Rohde et al., 2005). While Brouwer et al. (2017) derived COALS representations from a large corpus of newspaper text, we here derive them directly from the training data in order to exert more control over the resulting vectors. That is, our objective here is to arrive at distributed, partially overlapping semantic feature vectors, and not necessarily at feature vectors that reflect human similarity judgments (see Brouwer et al., 2017, for discussion). While these vectors could in principle be constructed by hand, the COALS method allows us to automatically derive them from our training sentences. Critically, an artifact of applying the COALS method to a data set of such small size, is that one may obtain identical vectors for two or more words. We mitigate this by concatenating the resulting COALS vectors with an identifier that assures that each word meaning vector is unique.

First, we computed a co-occurrence matrix using a 1-word window. We then converted the co-occurrence frequencies into pairwise correlations. Following the COALS procedure, we then discard negative correlations by setting them to zero, and we reduce the distance between weak and strong positive correlations by replacing them with their square root. Finally, as the training set contains 16 lexical items, we derived 16-dimensional binary word meaning vectors by replacing non-zero values with 1. To assure unique vectors for all words, the 16-dimensional vectors were concatenated with a 26-unit identifier containing two hot bits, resulting in 42-dimensional unique word meaning representations.

#### 2.2.3. Utterance Meaning Representations

Following Venhuizen et al. (2019a), the semantics associated with the training sentences presented to the model are derived from the Distributed Situation-state Space model (DSS, Frank et al., 2003, 2009; see also the formalization in terms of Distributional Formal Semantics described in Venhuizen et al., 2019c). In DSS, utterance meaning vectors are derived from a meaning space that defines co-occurrences of individual propositional meanings across a set of observations (formalized as formal semantic models in Venhuizen et al., 2019c). For the current meaning space, a set of propositions was generated using the predicates *enter(p,l), leave(p,l)*, and *go_to(p,g)*, in combination with arguments that identify a person (*p*), location (*l*), and goal (*g*) (see Table 1). In addition, the meaning space contains the unary predicates *entity* and *event* that assert the existence of referential entities and events, respectively, in the observations that constitute the meaning space: predicate names (*enter, leave*, and *go_to*) instantiate arguments for *event* propositions, and persons, locations and goals, together with a set of location-specific referents (*r*) instantiate arguments for *entity* propositions. This resulted in a total of 40 atomic propositions.

**Table 1:** Propositions described in the current meaning space and their arguments.

**Type**	**Variable**	**Instantiation**
proposition	–	enter(*p*,*l*), leave(*p*,*l*), go_to(*p*,*g*), entity(*p*), entity(*l*), entity(*g*), entity(*r*), event(enter), event(leave), event(go_to)
person	*p*	kevin
location	*l*	church, cinema, farm, school
goal (*church*)	*g*	bible
goal (*cinema*)	*g*	cash_register
goal (*farm*)	*g*	cows
goal (*school*)	*g*	classroom
goal	*g*	bus_stop, parking, toilet, tram
referent (*church*)	*r*	candle, hymn_book
referent (*cinema*)	*r*	popcorn_machine, seat
referent (*farm*)	*r*	farmer, pitchfork
referent (*school*)	*r*	teacher, rector

*In the first column, location names in brackets indicate that certain goals and referents are associated with particular locations, triggering presupposed entities (see text for details)*.

Based on this set of propositions P, a meaning space is constructed using an incremental, inference-driven probabilistic sampling algorithm (see Venhuizen et al., 2019c). The sampling algorithm uses a set of hard and probabilistic constraints to derive a set of models M that describe states-of-affairs in terms of combinations of propositions in P. Together, these models (i.e., observations) define a meaning space. The hard constraints used to derive the current meaning space restrict observations to describe a single *enter* or *leave* event, and at most one *go_to* event. In addition, predicates always co-occur with explicit referential introductions of each of their arguments and the denoted event [e.g., *enter(kevin,cinema)* always co-occurs with *entity(kevin), entity(cinema)*, and *event(enter)*]. Moreover, in order for the comprehension model to learn to associate locations to particular entities, certain propositions are constrained to always co-occur with certain presuppositions: locations always co-occur with their location-specific referents (selected based on the Cloze ratings from the DBC study), and each goal necessarily co-occurs with its associated location (as well as the associated presupposed referents). Probabilistically, the meaning space is constructed in such a way that goals occur more often with their related location than with any other location (following the plausibility ratings from the DBC study; see below).

Based on these constraints, we constructed a meaning space consisting of 3, 000 observations, which was reduced to 350 dimensions using the dimension selection algorithm described in Venhuizen et al. (2019a). The resulting meaning space defines meaning vectors for each of the propositions in P; the meaning of proposition p∈P is defined as the vector $\vec{v}\left(p\right)$, such that ${\vec{v}}_{i}\left(p\right)=1$ if *p* is true in model $M_{i}\in M$, and ${\vec{v}}_{i}\left(p\right)=0$ otherwise. These vectors can be compositionally combined in order to derive meaning vectors for logically complex expressions. In particular, the meaning of the conjunction between propositions *p* and *q* is defined as the point-wise multiplication of the meaning vectors $\vec{v}\left(p\right)$ and $\vec{v}\left(q\right)$: $\vec{v}\left(p\wedge q\right)=\vec{v}\left(p\right)\vec{v}\left(q\right)$ (Frank et al., 2003; Venhuizen et al., 2019a). The meaning vectors that are derived from the meaning space are also inherently probabilistic, as they define the fraction of models in which a proposition (or combination thereof) is true. More generally, given a meaning space of *n* observations, we can describe the probability of any point *a* in the meaning space (which may describe a proposition, a logical combination thereof, or any point in meaning space that cannot be directly expressed in terms of a logical combination of propositions) as follows (Frank et al., 2003; Venhuizen et al., 2019a):

(5) $$\begin{array}{l} P\left(a\right)=\frac{1}{n}\underset{i}{\sum }a_{i} \end{array}$$

Given the compositional nature of meaning vectors defined above, we can directly derive the conditional probability of any point in meaning space *a* given another point *b* in meaning space, that is, *P*(*a*|*b*) = *P*(*a*∧*b*)/*P*(*b*), which in turn can be used to derive the comprehension-centric notion of Surprisal (see Equation 4).

### 2.3. Training

#### 2.3.1. Training Sentences

To obtain model predictions for the conditions from the DBC study, we trained the model on a set of sentence-semantics pairs that were constructed based on a subset of the stimuli used for the DBC study (in German, but for clarity we here report the English equivalents). All sentences presented to the model are of the form “Kevin entered/left [loc] went_to [rel-tgt/unrel-tgt],” which are associated with the semantics *enter*(*kevin*,loc) ∧ *go_to*(*kevin*,rel-tgt/unrel-tgt) and *leave*(*kevin*,loc) ∧ *go_to*(*kevin*,rel-tgt/unrel-tgt), respectively. Table 2 shows the combinations of location (loc) and target (rel-tgt/unrel-tgt) that constitute sentences from the baseline/event-related condition (“Kevin entered/left [loc] went_to [rel-tgt]”) and the event-unrelated condition (“Kevin entered [loc] went_to [unrel-tgt]”). In addition, to balance plausibility across the *enter*/*leave* sentences, we also created a set of counterbalance sentences with plausible completions for the *leave* event, based on the Cloze completions from the DBC study (“Kevin left [loc] went_to [rel-tgt]”).

**Table 2:** Verb-Location-Target pairs used for constructing the training data.

verb	loc	rel-tgt	unrel-tgt
enter	cinema	cash_register	bible
enter	farm	cows	classroom
enter	school	classroom	cash_register
enter	church	bible	cows
leave	[loc]	bus_stop/parking/tram/toilet	–

*Related targets (rel-tgt) are used for constructing the baseline and event-related (and counterbalance) sentences, and Unrelated targets (unrel-tgt) are used for constructing the event-unrelated sentences (see text for details)*.

The model is taught that any combination of verb–location–target is in principle possible (following Brouwer et al., 2017), but that sentences from the baseline condition are more frequent than other *enter*–location–target combinations (13:1), and that counterbalance sentences are more frequent (4:1) than other *leave*–location–target combinations. This results in a total of 160 training sentences, with 64 unique semantics, half of which constitute *enter* sentences and the other half *leave* sentences. All locations occur equally often across the entire training set (40×), as well as all targets (20×). In terms of the probabilistic structure of the DSS meaning vectors derived for these sentences, the conjunctive semantics associated with the sentences from the baseline condition have a higher probability (M = 0.04, N = 4) than the semantics of both the event-related (M = 0.009, N = 4) and the event-unrelated (M = 0.005, N = 4) conditions.

#### 2.3.2. Training Procedure

We used bounded gradient descent (Rohde, 2002), a modification of the standard backpropagation algorithm (Rumelhart et al., 1986), to train the model. Moreover, following Brouwer et al. (2017), we trained the model in two stages. In the first stage, we trained the integration module only; that is, the entire model modulo the **input** and **retrieval** layers. The integration module is trained to map sequences of word meaning representations onto utterance meaning representations. The model was trained for 2, 000 epochs, using a momentum coefficient of 0.9 and a learning rate of 0.1, which was scaled down by 10% after every 500 epochs. In the second stage, the weights of the integration module are frozen, and the **input** and **retrieval** layer are added back into the model. The entire model is then trained to map sequences of word form representations onto utterance meaning representations. In this second stage, the model was again trained for 2, 000 epochs, with a momentum coefficient of 0.5 and a learning rate of 0.025 (which was again scaled down by 10% after every 500 epochs). To assure generalizability of our results, we trained 10 instances of the model, each with different initial weight matrices. After training, we evaluated the models in terms of mean squared error, output-target similarity, and overall comprehension performance. Overall, performance of the models was very good (mean squared error: M = 0.11; SD = 0.03, output-target similarity: M = 0.96; SD = 0.01; Recall@1 = 100%, comprehension score: M = 0.65; SD = 0.03).

## 3. Neurobehavioral Correlates of Surprisal

### 3.1. Modeling Predictions

**Figure 3:**
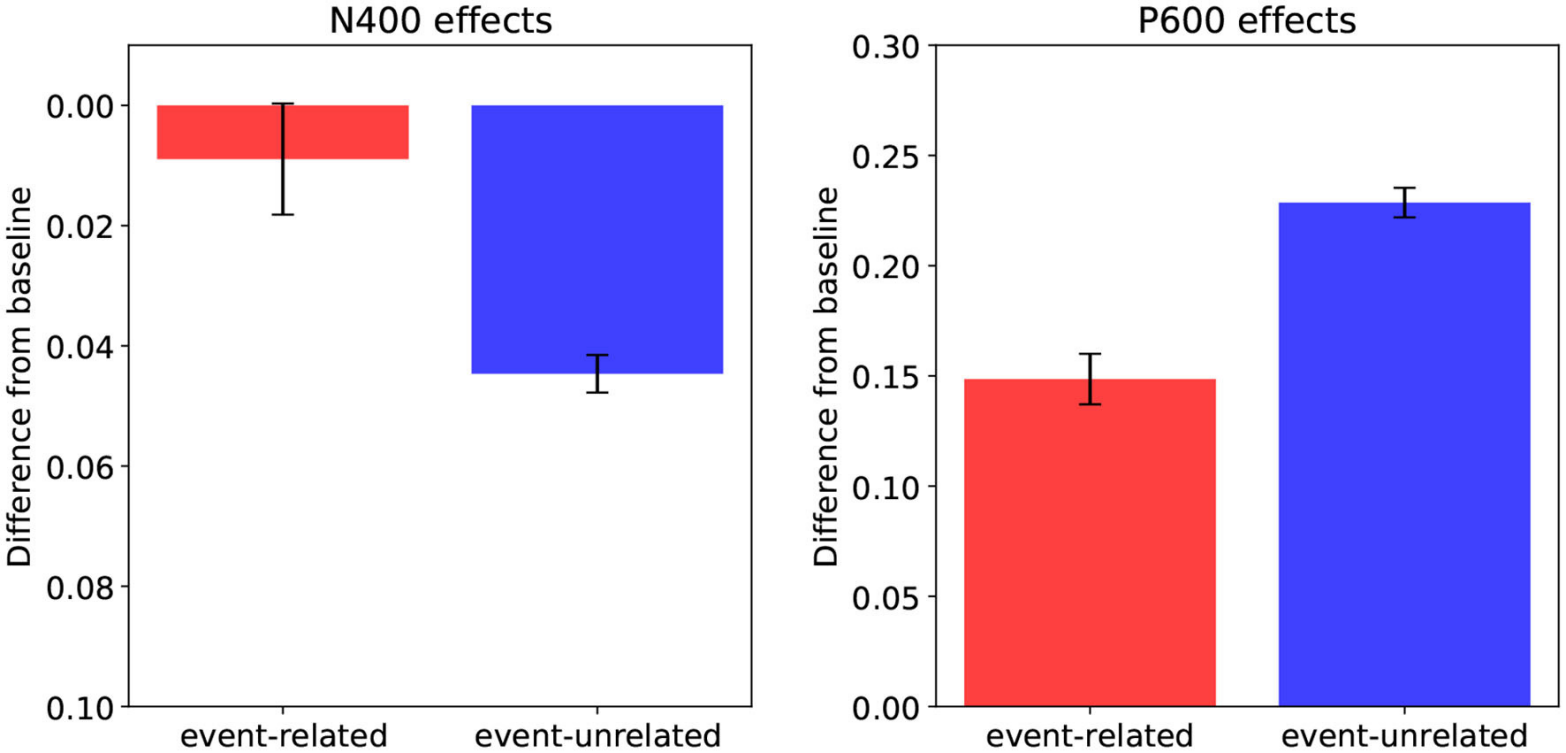
Model predictions: N400-effects (**left**, plotted downwards; see text) and P600-effects **(right)**, for the event-related condition relative to baseline, and for the event-unrelated condition relative to baseline. Error bars show standard errors.

To obtain model predictions, we computed N400, P600, and Surprisal estimates for the three conditions of the DBC experiment. Figure 3 shows the estimated N400 and P600 effects for the event-related relative to baseline contrast, and the event-unrelated relative to baseline contrast. While increased N400 and P600 estimates are positive distances in the **retrieval** and **integration** layers of the model, respectively, we plot the estimated N400-effects downward to signify the negative direction of the corresponding effects in the ERP signal. Note that the inputs and outputs of the retrieval and integration processes differ fundamentally and as consequence, the internal representations that the model develops at the **retrieval** and **integration** layers will also differ. Therefore, the absolute magnitudes of the N400 and P600 estimates should not be directly compared, and also do not directly map onto scalp-recorded voltages; that is, only the relative distances between the conditions in the **retrieval** and **integration** layers are of interest.

The predicted N400 estimates (Figure 3, left) show that while the model predicts a larger N400 amplitude for the event-unrelated condition relative to baseline, it predicts little to no difference between baseline and the event-related condition. Indeed, the N400 estimates pattern with the association manipulation, showing that a higher degree of association of a target word to its context leads to more facilitated retrieval of its meaning. The P600 estimates (Figure 3, right), in turn, reveal that relative to baseline, both the event-related and the event-unrelated condition produce larger estimated P600 amplitudes in the model. Here, the results pattern with the plausibility ratings and the Cloze probabilities. That is, the more implausible a target word is in a given context, and the lower its Cloze probability, the higher the P600 estimate it induces, reflecting increased effort in integrating its meaning into the unfolding utterance interpretation.

**Figure 4:**
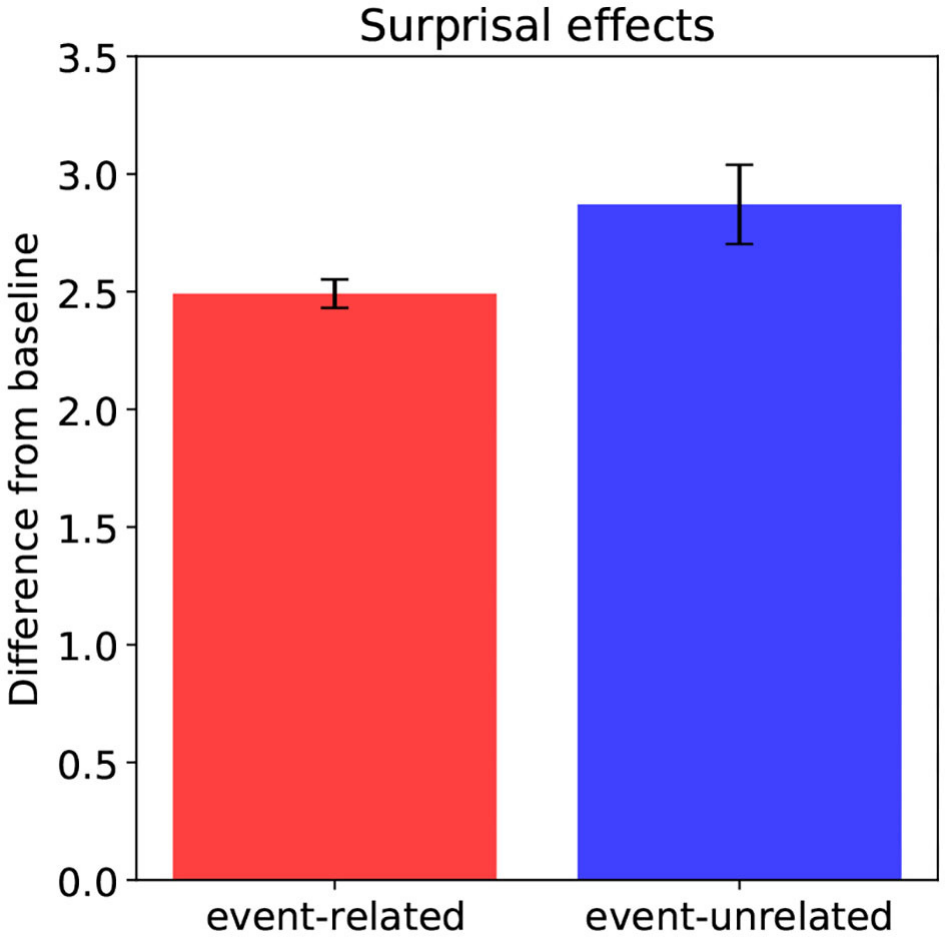
Model predictions: Surprisal effects for the event-related condition relative to baseline, and for the event-unrelated condition relative to baseline. Error bars show standard errors.

The Surprisal estimates (Figure 4) also follow the plausibility ratings and Cloze probabilities: the more implausible a word is in context, and the lower its Cloze probability, the higher its Surprisal according to the model. This means that integrating an implausible, unexpected word yields an interpretation—a point in situation-state space—that is improbable given the interpretation constructed prior to encountering it. Crucially, the Surprisal estimates clearly align with the P600 estimates, and not with the N400 estimates, suggesting a link between Surprisal and the P600. Indeed, while P600 amplitude in the model reflects the effort involved in updating the unfolding interpretation with the meaning contributed by the incoming word, that is, the work involved in actually traversing from one point to the next in situation-state space, Surprisal estimates reflect the likelihood of this traversal.

In sum, relative to baseline, the model predicts an N400-effect for the event-unrelated, but not for the event-related condition. The N400 estimates thus pattern with the association ratings. As for the P600 and Surprisal estimates, the model predicts an effect for both the event-related and the event-unrelated condition relative to baseline. Both the P600 and Surprisal estimates thus follow the plausibility ratings and Cloze probabilities.

### 3.2. Electrophysiological Results

**Figure 5:**
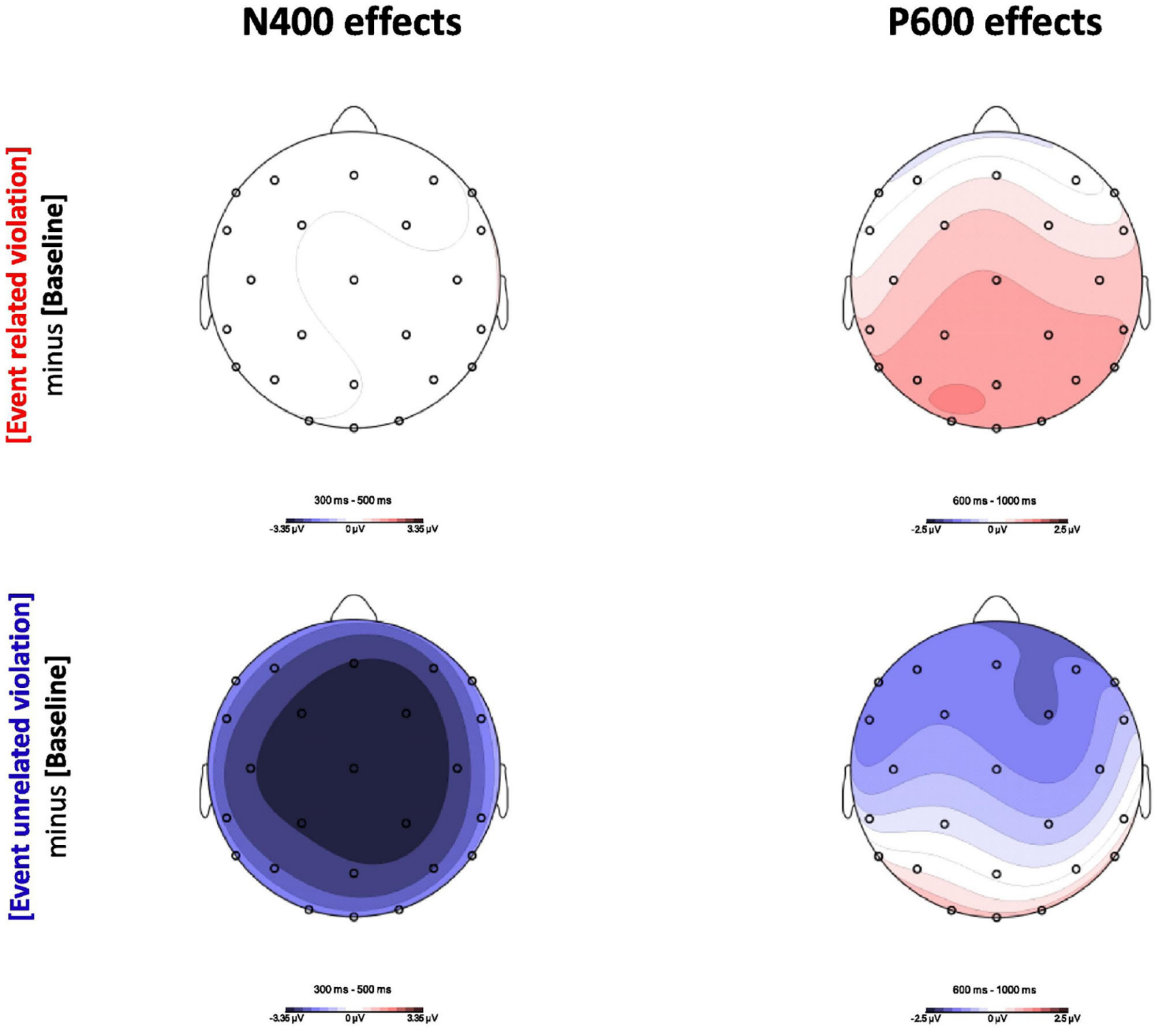
Topographic maps of the ERP effects in the N400 time window (300–500 ms, left column) and the P600 time window (600–1, 000 ms, right column). The upper panel shows the difference between the event-related condition and the baseline. The lower panel shows the difference between the event-unrelated condition and the baseline. Reproduced with permission (CC BY-NC-ND 4.0) from Delogu et al. (2019).

DBC report on the electrophysiological responses associated with the event-related and event-unrelated conditions. Figure 5 shows the ERP results in the N400 (300–500 ms, left column) and P600 (600–1, 000 ms, right column) time windows, for the event-related and event-unrelated conditions relative to baseline. The event-related condition, which only differs from baseline in plausibility, produced no difference in the N400 time window (top left), but a clear positive effect in the P600 time window (top right). The event-unrelated condition, in turn, which differs from baseline in both association and plausibility, produced a clear negative effect in the N400 time-window (bottom left), which sustained into P600 time window, albeit more frontally pronounced (bottom right). Indeed, while the overall pattern of results in the N400 time window support the view that association is manifest in N400 amplitude, which is in line with the predictions from the model, the results in the P600 time window are less clear. That is, while the results for the event-related condition support the view that plausibility is reflected in the P600, consistent with the model, the results for the event-unrelated condition seem to go against this.

Crucially, DBC argue that the P600 results may be reconciled if one factors in spatiotemporal overlap between the N400 and the P600; that is, they argue that P600 amplitude for the event-unrelated condition in the P600 time window is attenuated by spatiotemporal overlap with the N400. DBC substantiate this explanation by pointing out that—as would be predicted when spatiotemporal component overlap is at play—the broad negativity observed in the N400 time window becomes more frontally pronounced in the P600 time window, where a significant positivity arises at the occipital electrodes. This issue of spatiotemporal component overlap in interpreting ERP data is generally acknowledged (see Hagoort, 2003; Brouwer and Crocker, 2017, for discussions specific to language comprehenion), but as it affects the signal prior to recording, it presents a problem that is notoriously hard to mitigate; that is, given that the N400 and the P600 sum into a single scalp-recorded voltage, isolating their contribution requires a technique that allows for decomposing this voltage into its relevant constituent, latent voltages.

Brouwer et al. (2020) have recently shown that regression-based ERP (rERP) waveform estimation, as proposed by Smith and Kutas (2015a,b), allows for such a decomposition of scalp-recorded voltages. In an rERP analysis, linear regression models are fitted for each subject, time point, and electrode separately, using predictors that instantiate stimulus properties for each trial. Brouwer et al. (2020) derive an rERP analysis of the DBC data using *plausibility* and *association* as predictors. That is, for each subject, time point, and electrode, they fit the following linear regression model to the data:

(6) $$\begin{array}{l} y_{i}=\beta_{0}+\beta_{1}\text{plausibility}+\beta_{2}\text{association}+\epsilon_{i} \end{array}$$

where β_0_ is an intercept, β_1_ the slope for *plausibility* predictor, and β_2_ the slope for *association* predictor. For a given trial *i*, the predicted value *y*_*i*_ is the estimated voltage, the residual ϵ_*i*_ is the difference between the observed voltage and this estimate, and the predictors *plausibility* and *association* are set to their relevant values for the stimulus presented at this trial. Given a set of trials *y*_1_ … *y*_*n*_, the β coefficients are then fitted by minimizing total squared residuals ($\overset{n}{\underset{i}{\sum }}\epsilon_{i}^{2}$) across trials.

Using these fitted models, an rERP data set can be computed in which each observed voltage is replaced by an estimated voltage. Brouwer et al. (2020) show that the resultant rERP data set adequately mimics the observed ERP data, both in terms of residuals (by examining grand-average residuals for each electrode and time point) and in terms of variance (by subjecting the rERP data to the same statistical analysis as the ERP data; that is, by effectively treating it as a replication study). Crucially, as each estimated voltage is now a linear combination of *plausibility* and *association*, the individual contribution of one predictor can be isolated by neutralizing the other (e.g., by setting it to its mean value across trials). This allows us to obtain an clear view on what is going on in the N400 and P600 time-windows.

**Figure 6:**
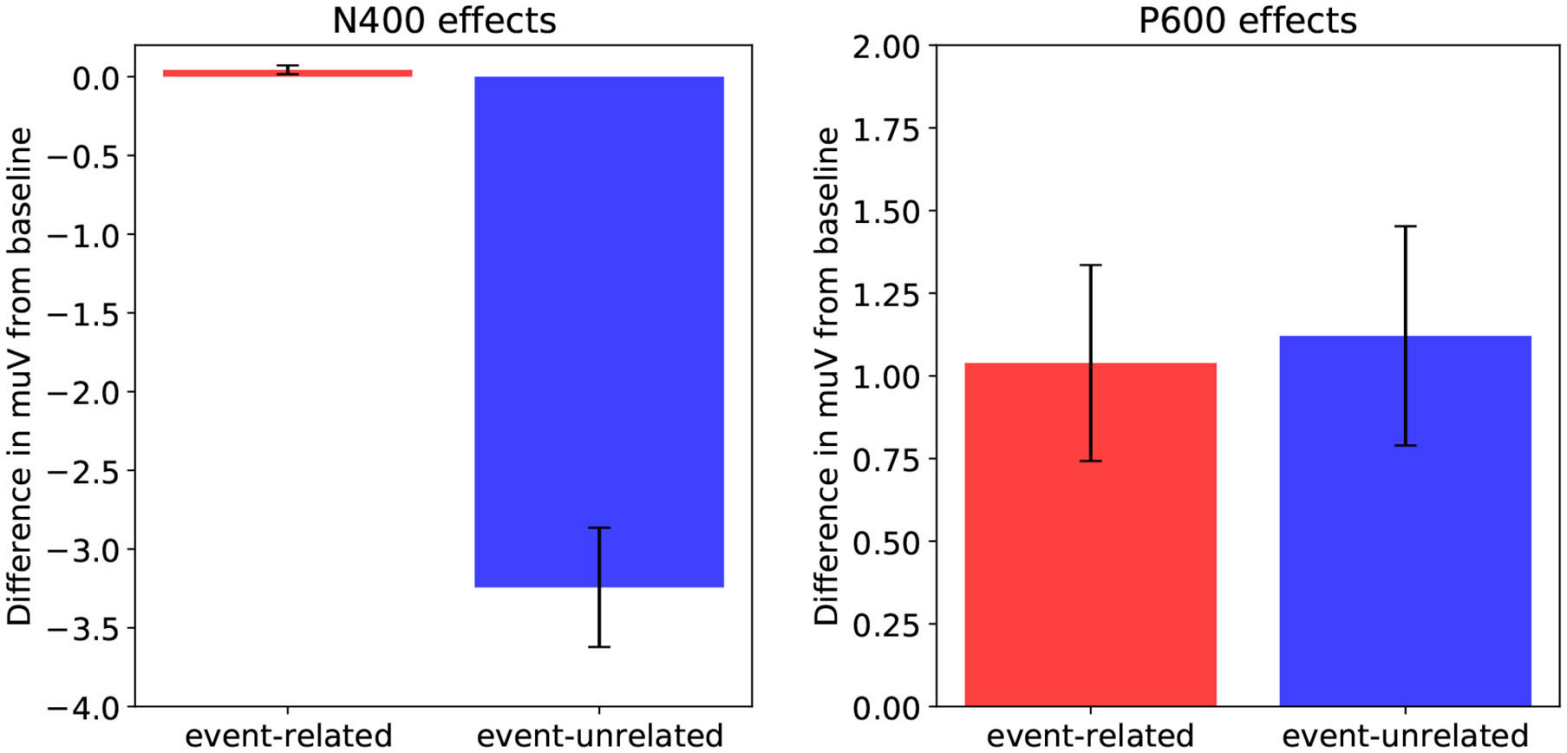
Effects as estimated using regression-based ERP (rERP) estimation: the isolated effects of association in the N400 time-window (300–500 ms, left), and the isolated effects of plausibility in the P600 time-window (600–1, 000 ms, right) for the event-related condition relative to baseline, and for the event-unrelated condition relative to baseline. Error bars show standard errors.

Starting with the N400 time window, we observe that the results align with the association manipulation. That is, we observe a difference between event-unrelated and baseline, which differ in association, and not between event-related and baseline, which do not differ in association. Moreover, as both the event-related and event-unrelated condition are more implausible than baseline, there is no possible constellation in which plausibility drives the N400, but gets attenuated in the event-related condition through association (as their is no difference in association between event-related and baseline). Finally, given that we do not observe a difference between event-related and baseline, plausibility seems to have little to no effect on the N400 results. Figure 6 (left) shows the N400-effects in the rERP data when the influence of *association* is isolated (by neutralizing *plausibility*). As in the ERPs, there is no difference between the event-related condition and baseline, while there is a large N400-effect for the event-unrelated condition relative baseline. Indeed, neutralizing the effect of *plausibility* has little effect on the results in the N400 time-window, confirming that the N400 results are driven by association.

As for the P600 time window, it is clear that the P600-effect for the event-related condition relative to baseline must be driven by plausibility, as these conditions do not differ in association. The question here, however, is how association and plausibility combine to explain the results for the event-unrelated condition relative to baseline. Figure 6 (right) show the P600-effects in the rERP data when the influence of *plausibility* is isolated (by neutralizing *association*). This shows the expected P600-effect for event-related relative to baseline, but critically, also a P600-effect for event-unrelated relative to baseline. Indeed, this suggests that the negativity that was observed for event-unrelated relative to baseline in the ERP data, can be explained by association and plausibility pulling in opposite directions, and association being the stronger force. Crucially, as association seems to drive the N400, and plausibility the P600, this thus suggests that the increase in P600 amplitude for the event-unrelated condition—which we revealed by isolating the effect of plausibility—is attenuated by spatiotemporal overlap with a sustained N400 driven by association.

In sum, when spatiotemporal component overlap between the N400 and the P600 is taken into account, the electrophysiological results of DBC align closely with the predictions of the model (compare Figures 3, 6): an N400-effect for event-unrelated relative to baseline, and a P600-effect for both the event-related and the event-unrelated conditions relative to baseline.

### 3.3. Behavioral Study

Surprisal has been typically linked to reading times (Levy, 2008). To investigate the behavioral cost associated with the implausible (and therefore higher in Surprisal) conditions from the study reported in DBC, and how this cost relates to the observed ERP responses, we have replicated the DBC study as a self-paced reading (SPR) experiment. Previous work investigating the effects of both plausibility and lexical association on reading times in sentence or discourse contexts has shown robust effects of plausibility, while the effects of lexical association are weaker and appear to be modulated by the global context (see Ledoux et al., 2006). For example, using eye-tracking, Camblin et al. (2007) found effects of discourse congruence on both the target and spillover regions of their stimuli, while effects of association were only observed in the target region for incongruent words. Moreover, Frank (2017) has argued that any effect of semantic relatedness on reading times may be due to a confound with word predictability. Based on these findings, we expect reading times to be mainly affected by plausibility on both the target and spillover regions. In particular, we expect longer reading times for critical words that are lexically associated with the preceding context but implausible, compared to associated and plausible targets.

#### 3.3.1. Method

##### 3.3.1.1. Participants

Thirty-one participants from Saarland University took part in the experiment. All had normal or corrected-to-normal vision, and none had participated in the DBC study. All were native German speakers, gave a written informed consent and were paid to take part in the experiment.

##### 3.3.1.2. Materials

The materials were the same as those used in the DBC study. There were 90 two-sentence discourses in German in three conditions (baseline, event-related implausible, event-unrelated implausible) intermixed with 90 filler passages. Experimental items and fillers were arranged in three counterbalanced lists (see, for details Delogu et al., 2019, p. 3–4).

##### 3.3.1.3. Procedure

The procedure was maintained as close as possible to the procedure in the ERP study by DBC. The context sentence in each pair was presented as a whole. Then a fixation cross appeared in the center of the screen. Participants had to press the space bar on the keyboard to proceed. Next the target sentence appeared word-by-word in the center of the screen. Participants controlled the rate of presentation of each word by pressing the space bar. At the end of each trial participants were asked to judge the plausibility of the mini-discourse by pressing one of two keys on the keyboard. The position of the plausible and implausible keys was counterbalanced across participants.

##### 3.3.1.4. Analysis

Statistical analyses were performed on two critical regions, the target word (*menu*) and a spillover region corresponding to the function word following the target (*und*)^2^. We present the results for the two regions separately and also for the two regions combined into a single one, in order to decrease noise. Prior to statistical analysis, reading times (RTs) shorter than 80 ms and longer than 2, 500 ms were discarded for each region (for the combined region, we discarded RTs shorter than 160 ms and longer than 5, 000 ms)^3^. Linear mixed-effects regression models (LMMs) were fitted to log-transformed RTs, with condition (three levels: baseline, event-related implausible, event-unrelated implausible), as the fixed effects, and participants and items as random effects. The condition variable was effect-coded. Contrasts were used to compare the two implausible conditions with the baseline (effect of plausibility) and the event-related with the event-unrelated conditions (effect of association in the implausible conditions). In evaluating the models, we started with the maximal structure of random effects, which included random intercepts and slopes for both subjects and items. The random structures were then simplified by progressively excluding the effects explaining the least amount of variability in the model (following Bates et al., 2015). For each statistical model, we report effect coefficients (β), standard errors (SEs), and *t*-values (t). If the absolute value of t exceeded 2.5, the coefficient was judged to be significant.

#### 3.3.2. Results

##### 3.3.2.1. Plausibility Judgements

Participants judged the baseline condition to be more plausible than the event-related and the event-unrelated conditions (baseline: 91%; event-related: 24%; event-unrelated: 8%). These results closely mirror the offline plausibility ratings and online judgments reported in the DBC study.

##### 3.3.2.2. Reading Times

**Figure 7:**
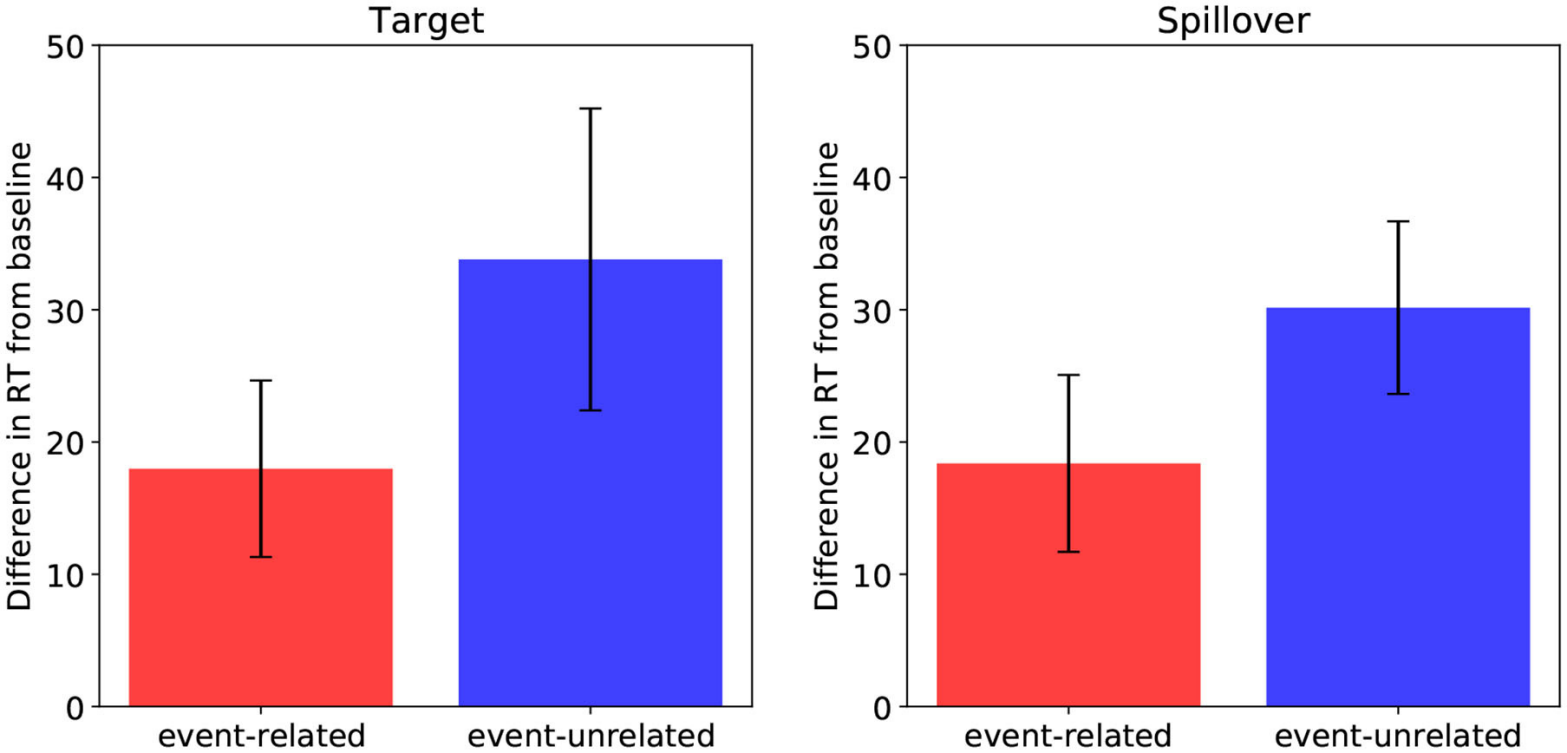
Self-paced reading times (RTs) effects in the target region **(left)** and the spillover region **(right)**, for the event-related condition relative to baseline, and for the event-unrelated condition relative to baseline. Error bars show standard errors.

Figure 7 shows the results^4^. At the target word, participants were slower to read both in the event-related (M = 434.8 ms, SD = 182.9) and the event-unrelated (M = 450.6 ms, SD = 221.8) conditions compared to the baseline (M = 416.8 ms, SD = 175.3). The results of the LMM analysis revealed a significant effect of plausibility (β = 0.035, SE = 0.013, t = 2.64) and no difference between the two implausible conditions (β = 0.018, SE = 0.019, t = 0.985).

The same reading time pattern emerged at the spillover word. Participants were slower to read both in the event-related (M = 377.9 ms, SD = 89.0) and the event-unrelated (M = 389.7 ms, SD = 95.5) conditions compared to the baseline (M = 359.5 ms, SD = 84.5). While the effect of plausibility was significant (β = 0.05, SE = 0.013, t = 3.960), the difference between the event-related and the event-unrelated conditions was not (β = 0.022, SE = 0.014, t = 1.61).

LMMs on the region including both the target and the spillover word showed an effect of plausibility (β = 0.051, SE = 0.012, t = 4.299) and a marginal difference between the two implausible conditions (β = 0.028, SE = 0.014, t = 2.004).

To summarize, in the analysis of the target and spillover regions, both the event-related and the event-unrelated conditions took longer to read than the baseline, suggesting that reading times were sensitive to plausibility rather than association. However, the event-unrelated condition was numerically slower than the event-related condition, possibly suggesting an additive effect of association and plausibility. To further investigate the relative contribution of these factors in predicting reading times, we fitted LMMs to log-transformed RTs in the merged target and spillover region, with plausibility and association ratings (and their interaction) as continuous predictors, and participants and items as random factors. Both plausibility and association were inverted and z-transformed prior to analysis (see Brouwer et al., 2020). Model selection procedure was the same as in the previous analysis. There was no effect of association (β = 0.005, SE = 0.010, t = 0.49), and no interaction of association and plausibility (β = 0.006, SE = 0.011, t = 0.53). Plausibility, however, significantly predicted reading times in this region (β = 0.025, SE = 0.009, t = 2.717). Thus, plausibility appears to be a more robust predictor of reading times than association in the target and spillover region.

In sum, the behavioral results show increased reading times for both the event-related and event-unrelated condition relative to baseline, and no effect of association, consistent with previous findings showing a reading time cost for implausible targets (e.g., Ledoux et al., 2006). These results pattern with the P600 results from DBC (compare Figure 7 to Figure 6), as well as with the P600 and Surprisal estimates from the model (compare Figure 7 to Figures 3, 4).

## 4. Discussion

We have presented a neurocomputational model of incremental, word-by-word language comprehension that produces N400, P600, and Surprisal estimates for each word. In this model, which integrates the neurocomputational model of the Retrieval-Integration account (Brouwer et al., 2017) with a “comprehension-centric” model of Surprisal (Venhuizen et al., 2019a), N400 amplitude is hypothesized to reflect the effort involved in the context-dependent retrieval of word meaning, P600 amplitude is hypothesized to index the work required to integrate this retrieved word meaning into the unfolding utterance interpretation, and Surprisal is taken to reflect the likelihood of the resultant interpretation, given the interpretation prior to integrating the meaning contributed by the incoming word. We set out to test a key prediction of the model: The P600, and not the N400, indexes “comprehension-centric” Surprisal. To investigate this link, we obtained model predictions for a recent study by Delogu et al. (2019, DBC), which directly investigated the electrophysiological correlates of plausibility-induced Surprisal. We found that—when spatiotemporal overlap between the empirically observed N400 and P600 is taken into account—the predictions of the model closely align with the empirical ERP data, showing that while the N400 is driven by association between a target word and its context, plausibility drives the P600. Further, to assess the alignment of the Surprisal estimates of the model with behavioral indices of processing difficulty, we presented the results from a self-paced reading replication of the DBC study. These empirical results again align closely with the model predictions, showing increases in reading times that are predominantly driven by plausibility. Taken together, our results thus support the conclusion that the P600 is an index of “comprehension-centric” Surprisal.

While we have focused on plausibility-induced semantic Surprisal, this conclusion is consistent with the proposal that the P600 is an overarching index of compositional semantic processes (Brouwer et al., 2012), which is sensitive to syntax (e.g., Osterhout and Holcomb, 1992; Hagoort et al., 1993; Gouvea et al., 2010), semantics (e.g., Kutas and Hillyard, 1980; Kolk et al., 2003; Hoeks et al., 2004), and pragmatics (e.g., Burkhardt, 2006; van Berkum et al., 2007; Dimitrova et al., 2012). Moreover, by establishing a link between the P600 and expectancy, as quantified through Surprisal, an interesting question arises, namely if the P600 is indeed an instance of the P300, and in particular of the late P3b subcomponent that has been shown to be sensitive to the detection of salient “oddball” stimuli (for recent discussion, see Sassenhagen and Fiebach, 2019; Leckey and Federmeier, 2020). On the one hand, the proposed link between the P600 and expectancy may be tentatively be taken to suggest that the integrative processes underlying this component are similar to the hypothesized context-updating mechanisms underlying the P300 (Donchin and Coles, 1988). On the other hand, the P300 is strongly dependent on task-demands, and while the P600 is sensitive to the task at hand, the presence of an explicit task it not a prerequisite for its elicitation (Kolk et al., 2003). Hence, while the “P600-as-P3 hypothesis” (Sassenhagen et al., 2014) poses interesting question, our results do not further elucidate this relationship.

Importantly, the conclusion that the P600 indexes comprehension-centric Surprisal is fully consistent with results showing a reliable correlation between Surprisal and the N400 (e.g., Frank et al., 2015, who employ word Surprisal estimates derived from a language model). In fact, it follows from the architecture of the model that the unfolding interpretation should influence the retrieval of word meaning—which modulates the N400 estimates—through lexical and contextual priming (see Kutas and Federmeier, 2000; Lau et al., 2008; van Berkum, 2009; Brouwer et al., 2012; Brouwer and Hoeks, 2013, for detailed discussions on how these factors may influence retrieval). Indeed, the N400 is effectively a function of the degree to which the memory system anticipates the conceptual knowledge associated with an incoming word, and in general, anticipation in the memory system tends to correlate with the expectancy of a word, as quantified through its Cloze probability (Kutas et al., 1984; Thornhill and Van Petten, 2012). In these cases, N400 amplitude patterns with interpretation-level Surprisal, but is not a direct reflection of it. Crucially, studies such as those by DBC underline this indirectness, as they show that the semantic association of a target word to its context can overrule its unexpectedness, thereby producing no difference between expected and unexpected targets in the N400; also see the literature on Semantic Illusions (e.g., Kuperberg, 2007; Bornkessel-Schlesewsky and Schlesewsky, 2008; Brouwer et al., 2012, for reviews). It should be noted, however, that unlike in many of the Semantic Illusion studies, the DBC study rules out an explanation in which the absence of an N400-effect for unexpected, but associated targets is due to “shallow” integrative processing—as assumed in models in which the N400 is itself a direct index of integrative semantic processing (e.g., Rabovsky et al., 2018)—because the robust P600-effect for this condition, as well as high accuracy in behavioral implausibility judgments, show that comprehenders are explicitly aware of the unexpectedness of the target (see also Sanford et al., 2011). Further, given that the target sentences of the DBC stimuli were globally and locally unambiguous, this observed P600-effect cannot be explained by models that attribute the increase in P600 amplitude to index syntactic repair or reanalysis (e.g., Fitz and Chang, 2019).

We have qualitatively established the P600 as a direct index of “comprehension-centric” Surprisal by showing that its estimated amplitude increases in response to surprising, implausible target words, relative to unsurprising, plausible ones. An open question remains if the P600 is also a quantitative index of Surprisal; that is, if its amplitude is sensitive to expectancy in a graded manner. The experiment by DBC was not designed to address this question. We do observe, however, in both the electrophysiological and the behavioral results that the event-related condition at least numerically incurs less processing difficulty than the event-unrelated condition. Indeed, this is in line with the offline plausibility ratings and Cloze ratings, in which the event-related condition is rated as more plausible and expected than the event-unrelated condition, respectively. While this may suggest a graded difference in Surprisal between these conditions, we believe these ratings to be confounded by association; that is, in the event-related condition, the strong semantic association of a target word to its context, leads people to judge them as slightly more plausible, than the unassociated, implausible target words in the event-unrelated condition.

Interestingly, however, the model predicts the same graded pattern, both in its P600 estimates and in its Surprisal estimates, as observed in the empirical data. Crucially, in constructing the meaning space—from which the utterance meaning representations that the model recovers in processing are derived—we did not explicitly induce any probabilistic difference between the semantics associated with the two implausible conditions. Yet, we do observe a difference in that the semantics associated with the event-related sentences are slightly more probable than the semantics associated with the event-unrelated sentences. This difference can be explained by the structure of the meaning space, which is defined in terms of probabilistic co-occurrences. Indeed, given that the baseline and event-related condition share many of the same presuppositions, as instantiated by *entity* predicate (see above), their semantics occupy parts of the same region of the overall meaning space. The event-unrelated semantics, by contrast, trigger a different set of presuppositions, thereby constituting a different part of the meaning space. As during processing the model navigates the meaning space on a word-by-word basis, this spatial organization directly affects its behavior, as reflected in its P600 and Surprisal estimates; that is, the target word in event-unrelated sentences triggers a larger transition in meaning space than the target word in event-related sentences, thereby explaining the difference in P600 and Surprisal estimates. Hence, it is the presence of referential presuppositions, which serve to associate specific targets with specific contexts, that explains the graded pattern in the model. On a speculative note, the model thus effectively predicts plausibility to be confounded with association, which numerically aligns with the offline ratings and empirical results.

In sum, while our results support a qualitative link between Surprisal and the P600, it remains an open question if this extends to a quantitative one, in that, like reading times, the P600 is sensitive to expectancy in a graded manner. Given the issue of spatiotemporal component overlap, however, addressing this question may be challenging, as manipulating expectancy in a graded manner may also yield graded N400 results, thereby rendering it non-transparent what is going on in the P600 (e.g., see Thornhill and Van Petten, 2012). In future work, this can be addressed by using rERP analyses, which allow for disentangling the N400 and the P600 in space and time, on results from co-registered reading time and ERP studies.

## 5. Conclusion

We have presented a neurocomputational model of incremental, word-by-word language comprehension that produces N400, P600, and “comprehension-centric” Surprisal estimates at each word in a sentence. In the model, estimated N400 amplitude reflects the effort involved in the contextualized retrieval of the meaning of an incoming word, while estimated P600 amplitude indexes the effort involved in integrating this retrieved word meaning into the unfolding utterance interpretation. Surprisal estimates, in turn, reflect the likelihood of an updated interpretation, given the interpretation prior to updating it. By testing it on an experimental design that directly tests “world-knowledge”-induced Surprisal, we have shown that the predictions of the model align with empirical electrophysiological results—when spatiotemporal component overlap between the N400 and P600 is taken into account—as well as with behavioral reading times. We find a close relationship between Surprisal, which we take to be reflected by reading times, and P600 amplitude, thereby supporting the interpretation of the P600 as the ERP component that indexes “comprehension-centric” Surprisal. Future work must determine if this link is only qualitative, or if it also holds quantitatively, in that the P600, like reading times, is sensitive to graded manipulations of expectancy. Overall, we believe that this theory-driven linkage of electrophysiological and behavioral correlates of processing difficulty, through explicit neurocomputational modeling, provides an important step toward an integrated neurobehavioral theory of language comprehension.

## Data Availability Statement

The ERP and reading time data is available at: https://github.com/hbrouwer/dbc2019rerps.

## Ethics Statement

The studies involving human participants were approved by Deutsche Gesellschaft für Sprache (DGfS). The participants provided their written informed consent to participate in this study.

## Author Contributions

HB and NV conducted the computational modeling. FD conducted data analysis. All authors contributed equally to the writing of the manuscript.

## Conflict of Interest

The authors declare that the research was conducted in the absence of any commercial or financial relationships that could be construed as a potential conflict of interest.

## Footnotes

^1^Linking hypotheses such as these, between model behavior and the electrophysiological signal, are also known as “synthetic ERPs” (Barrès et al., 2013, see also beim Graben et al., 2008; Crocker et al., 2010; Rabovsky et al., 2018; Fitz and Chang, 2019, among others).

^2^The precritical region did not show any significant difference between conditions.

^3^The reading time data is available at: https://github.com/hbrouwer/dbc2019rerps.

^4^We did not exclude trials from the analyses on the basis of the results from the plausibility judgments.
